# Vividly coloured poppy flowers due to dense pigmentation and strong scattering in thin petals

**DOI:** 10.1007/s00359-018-01313-1

**Published:** 2019-01-28

**Authors:** Casper J. van der Kooi, Doekele G. Stavenga

**Affiliations:** 1 Groningen Institute for Evolutionary Life Sciences, University of Groningen, 9747 AG Groningen, The Netherlands; 2 Computational Physics, Zernike Institute for Advanced Materials, University of Groningen, 9747 AG Groningen, The Netherlands

**Keywords:** *Papaver*, Pollination, Anthocyanin, Bee vision, Reflectance

## Abstract

The flowers of poppies (Papaveraceae) exhibit bright colours, despite their thin and floppy petals. We investigated the optical properties of flowers of *Papaver rhoeas, P. dubium, Meconopsis cambrica* and *Argemone polyanthemos* using a combined approach of anatomy, spectrophotometry and optical modelling. The petals of *Papaver* flowers are composed of only three cell layers, an upper and lower epidermal layer, which are densely filled with pigment, and an unpigmented mesophyll layer. Dense pigmentation together with strong scattering structures, composed of serpentine cell walls and air cavities, cause the striking poppy colours. We discuss how various aspects of the optical signal contribute to the flower’s visibility to pollinators.

**Electronic supplementary material:**

The online version of this article (10.1007/s00359-018-01313-1) contains supplementary material, which is available to authorized users.

## Introduction

Flowers have been called sensory billboards (sensu Raguso 2004), because they feature numerous traits to entice pollinators. The bewildering diversity in floral colours is considered to have evolved with respect to the visual perception of their pollinators (e.g., Barth 1991; Chittka and Menzel 1992; Dyer et al. 2012; Muchhala et al. 2014; Shrestha et al. 2016). The colours of flowers are due to two basic optical principles: (1) reflection and scattering of light by the floral structures, and (2) selective absorption in a specific wavelength range by floral pigments (van der Kooi et al. 2016, 2019). Scattering occurs because petals consist of media with different refractive indices, such as cell walls, air cavities, water-filled vacuoles, and thus incident light is reflected and scattered at their interfaces (Kay et al. 1981; Kevan and Backhaus 1998; Lee 2007; Vignolini et al. 2012; van der Kooi et al. 2014). Pigments absorbing in a specific wavelength range filter the light flux travelling inside the petal, giving the flower a certain hue. For example, blue-absorbing carotenoids create yellow colours, and blue–green-absorbing anthocyanins create red colours. Though the chemistry, molecular synthesis and evolution of floral pigments have been extensively studied (e.g., Koes et al. 1994; Mol et al. 1998; Grotewold 2006; Rausher 2008; Hopkins and Rausher 2011; Zhao and Tao 2015; Sheehan et al. 2016), much remains unknown about how light propagates in a flower and how backscattering structures and pigments are tuned.

We recently studied the pigmentation and scattering properties of 39 species of flower, and found that flowers of the common poppy, *Papaver rhoeas*, are exceptional in various ways. *P. rhoeas* has fairly large and exceedingly thin petals, yet they are deeply coloured and are relatively strong scatterers (van der Kooi et al. 2016). These findings raise the question as to the anatomical details of *P. rhoeas* flowers that cause the extreme optical characteristics, and whether related species share similar properties.

Poppies are a group of genera in the subfamily Papaveroideae of the Papaveraceae, which is an early diverging eudicot family. They include species of *Papaver, Meconopsis*, and *Eschscholtzia*, and comprise many iconic species well-known for their showy flowers. Several of these species, such as *P. rhoeas, P. somniferum* (opium poppy), *Meconopsis grandis* (Himalayan poppy) and *Eschscholtzia californica* (California poppy) are popular ornamental and garden plants. For a few species, the characteristics of their flower colours have been studied in some detail. For example, the chemistry and vacuolar pH have been studied for blue-flowered *Meconopsis* species (Yoshida et al. 2006). *E. californica* has been investigated because of its pigmentation via the specific pigment eschscholtzxanthin (e.g., Strain 1938) and because of its ultrastructure (Wilts et al. 2018).

Here, we investigate the flower colours of *P. rhoeas, P. dubium* (long-headed poppy), the closely related *Meconopsis cambrica* (Welsh poppy), which has both yellow and orange colour morphs, and *Argemone polyanthemos* (crested prickly poppy). Using photography, spectrophotometry, optical modelling, and various anatomical techniques, we show that a high pigment content together with scattering air holes cause the typical coloration of these thin flowers. We discuss our results in context of the plant’s ecology and visual ecology of their pollinators.

## Materials and methods

### Plant material and photography

All flower samples were obtained from road sides and meadows around the campus of the University of Groningen, except for flowers of *A. polyanthemos*, which were grown from seeds purchased at De Bolster, Epe, The Netherlands. Flowers were photographed with a digital camera (Nikon D70) equipped with an F Micro-Nikkor macro objective (60 mm, f2.8, Nikon, Tokyo, Japan). Petal details were photographed with an Olympus DP70 digital camera mounted on an Olympus SZX16 stereomicroscope (Olympus, Tokyo, Japan), or with a Zeiss Universal microscope (Zeiss, Oberkochen, Germany) equipped with a DCM50 camera (Mueller Optronic, Erfurt, Germany).

### Spectrophotometry

Reflectance and transmittance spectra of petals were measured with an integrating sphere (for technical details and measurement procedures, see Stavenga and van der Kooi 2016). The sphere’s measurement area (its aperture) is approximately 5 mm. In contrast to the commonly used reflectance probe, an integrating sphere allows measuring the absolute amount of backscattering as well as the modulation of the spectrum (for further discussion on the sphere and probe, see Vukusic and Stavenga 2009). The pigment absorbance spectrum was measured with a microspectrophotometer (MSP, see Stavenga and van der Kooi 2016). A piece of flower was immersed in water; the measurement area was a square with side length ~ 10 µm. We subtracted the long-wavelength absorbance, which is due to scattering, to estimate the contribution of the absorbing pigments. Measurements were taken from flowers of at least three individuals per species.

We also investigated the angle dependence of the reflectance by illuminating the flat surface of a *P. rhoeas* petal with a rotatable fibre and collecting the reflected light with another rotatable fibre, positioned at the mirror angle of the illumination. The latter fibre was fitted with a polarizer, which allowed measurement of reflectance spectra as a function of angle of light incidence for both TE (transverse electric) and TM (transverse magnetic) light.

### Anatomy

The thickness of the petals was measured on pieces placed in between two cover slips with a thickness gauge. We measured the thickness for each flower five times on a transect from the proximal to distal part of a petal, for 3–5 individuals per species (Table 1). The pigment distribution of the flowers was examined via transverse sections of flower pieces. Flower pieces were embedded in 6% agarose solution at a temperature of approximately 55 °C, i.e., close to the temperature of solidification. Transverse sections were cut using a sharp razor blade and immediately examined with the Zeiss Universal microscope. Satisfactory results could be obtained only for *M. cambrica*. Essentially, the same distribution was observed for the other studied species, but the very thin *Papaver* petals precluded obtaining presentably clear pictures.

**Table 1:** Thickness measurements (in µm)

Species	No. individual plants	Min	Max	Mean	SD
*Papaver rhoeas*	5	56	266	108	62
*Papaver dubium*	5	48	319	101	65
*Meconopsis cambrica* yellow	5	74	279	157	65
*Meconopsis cambrica* orange	5	73	289	142	29
*Argemone polyanthemos*	3	131	407	266	89

Per petal, thicknesses were measured five times on a transect from proximal to distal (see “Materials and methods”)

### Optical modelling

We used the measured reflectance and transmittance spectra of the petals to calculate the petals’ overall absorption and scattering parameters. A flower can be considered as a stack of layers, where different layers have specific scattering and pigmentation properties (van der Kooi et al. 2016). Light microscopical observations as well as thickness measurements on *P. rhoeas* and *P. dubium* flowers suggested that the flowers are composed of only a few cell layers (Table 1). In line with observations by Kay et al. (1981), our anatomical investigations showed that the petals consist of three main layers, i.e., a pigmented upper and lower epidermis, with an unpigmented (mesophyll) layer in between. We hence deployed the optical model that we developed for understanding the colours of the Chilean bellflower, *Nolana paradoxa*, which combines the Kubelka–Munk theory for absorbing and scattering media with a layer-stack light-propagation model (Stavenga and van der Kooi 2016). Using measured transmittance spectra as well as adaxial and abaxial reflectance spectra, we could calculate the absorbance parameter K* = *Kd* and scattering parameter S* = *Sd*, where *K* and *S* are the absorption and scattering coefficients and *d* the petal thickness. The modelling showed that asymmetric petals consisting of one pigmented and one unpigmented layer cause very different adaxial and abaxial colours. However, identical adaxial and abaxial reflectance spectra result when the petal is homogeneously pigmented or symmetrically organized into three layers, and the pigment is equally distributed in the two peripheral layers. Using the calculated absorption and scattering parameters in a calculation of the transmittance and reflectance spectra for a symmetrical, three-layer case yielded spectra virtually identical to the experimentally measured spectra. Finally, the transmittance of a homogeneously pigmented layer with absorption coefficient *K* and thickness *d* is calculated as:

1 $$T={\text{ exp}}\left( { - {K^*}} \right),$$

so that the absorbance is

2 $$D{\text{ }}= - {\text{lo}}{{\text{g}}_{{\text{1}}0}}\left( T \right)={\text{ }}g{K^*}=K^{\prime},$$

with *g* = log10(*e*) = 0.4343.

### Vision modelling

We investigated the visibility of the flowers with a pollinator-subjective view for known poppy pollinators, i.e., honey bees. We analysed the measured reflectance spectra under D65 ambient light against a green leaf background as before (van der Kooi et al. 2016), with two well-established vision models, i.e., the color hexagon model (Chittka 1992) and the receptor noise-limited model (Vorobyev and Osorio 1998). Both models yield values that correlate with the flower contrast as perceived by bees. Green contrast was calculated as per Spaethe et al. (2001).

## Results

### Poppies vary in coloration and pigmentation

**Fig. 1:**
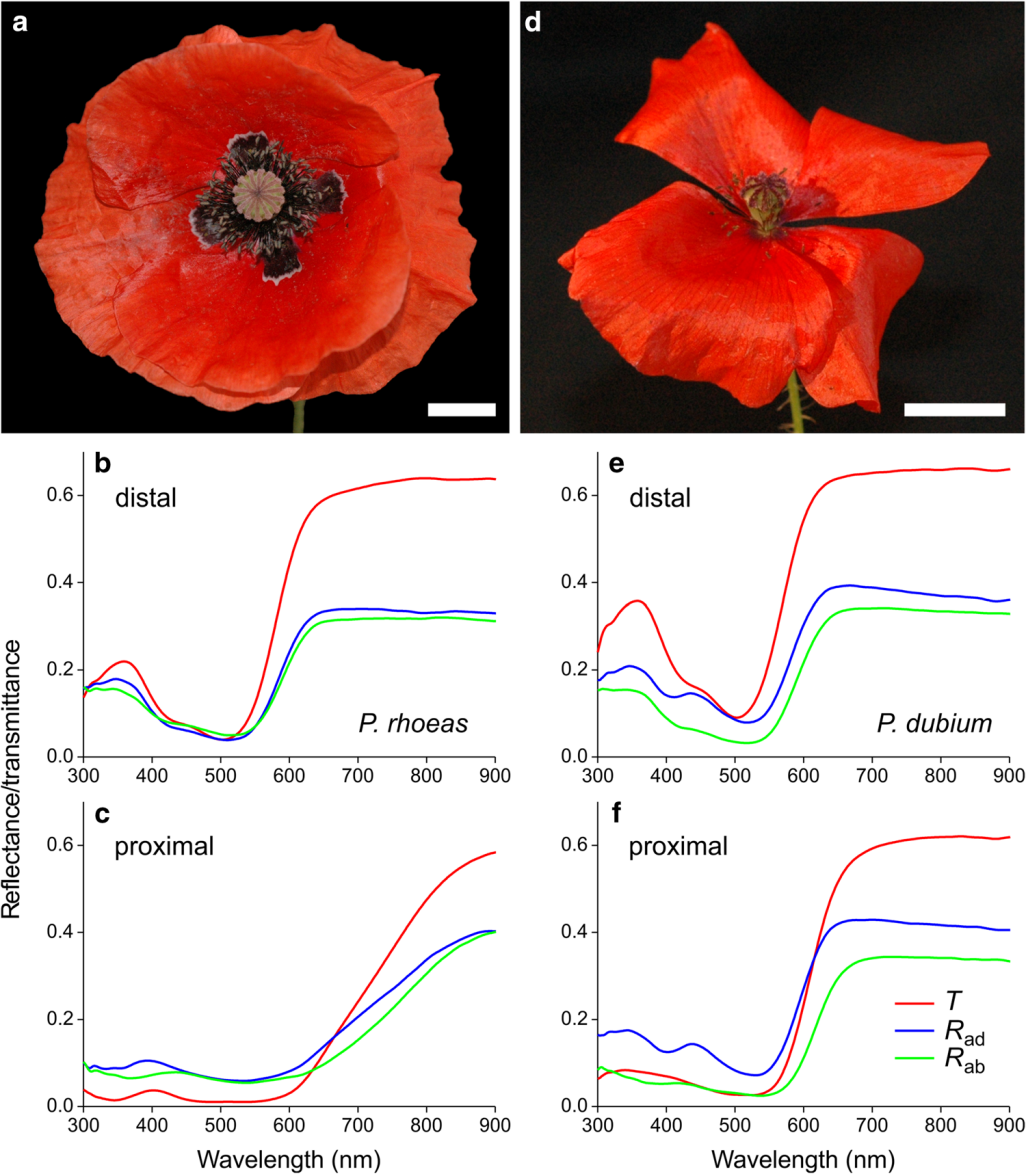
Habitus pictures and spectra of two exemplary poppies. **a**–**c***P. rhoeas*. **d**–**f***P. dubium*. Transmittance (*T*) and adaxial (*R*_ad_) and abaxial (*R*_ab_) reflectance spectra of distal (**b**–**e**) and proximal (**c**–**f**) petal areas. Scale bars (**a, b**) 1 cm

The common poppy (*P. rhoeas*) features typically red flowers, with at the petal base often a distinctly black area bordered by a thin white line (Fig. 1a). The reflectance and transmittance values at very long wavelengths (> 900 nm) can be used to quantify the backscattering by the petal, because in that wavelength range absorption by pigments is negligible (see also van der Kooi et al. 2016). For both the distal and proximal areas, the transmittance is ~ 0.65 and the reflectance ~ 0.35 (Fig. 1), meaning that the petals scatter approximately 35% of incident light. Even in the case of the proximal (base) area, which is deeply black coloured, the reflectance and transmittance curves plateau at similar amplitude in the long wavelength range. Although the long-wavelength reflectance of ~ 0.35 may seem to be in conflict with the blackness of the base area, the reflectance in the visible wavelength range is small and the gradual increase in reflectance at wavelengths > 600 nm is too small to give a colourful signal. The low transmittance and reflectance at shorter wavelengths must be due to strongly light-absorbing pigments. The different slopes of the distal and proximal spectra at wavelengths > 550 nm indicate different pigments. In the ultraviolet wavelength range, the transmittance and reflectance of the distal area is distinctly higher than the corresponding value for the proximal part, which also indicates the presence of a different pigment.

**Fig. 2:**
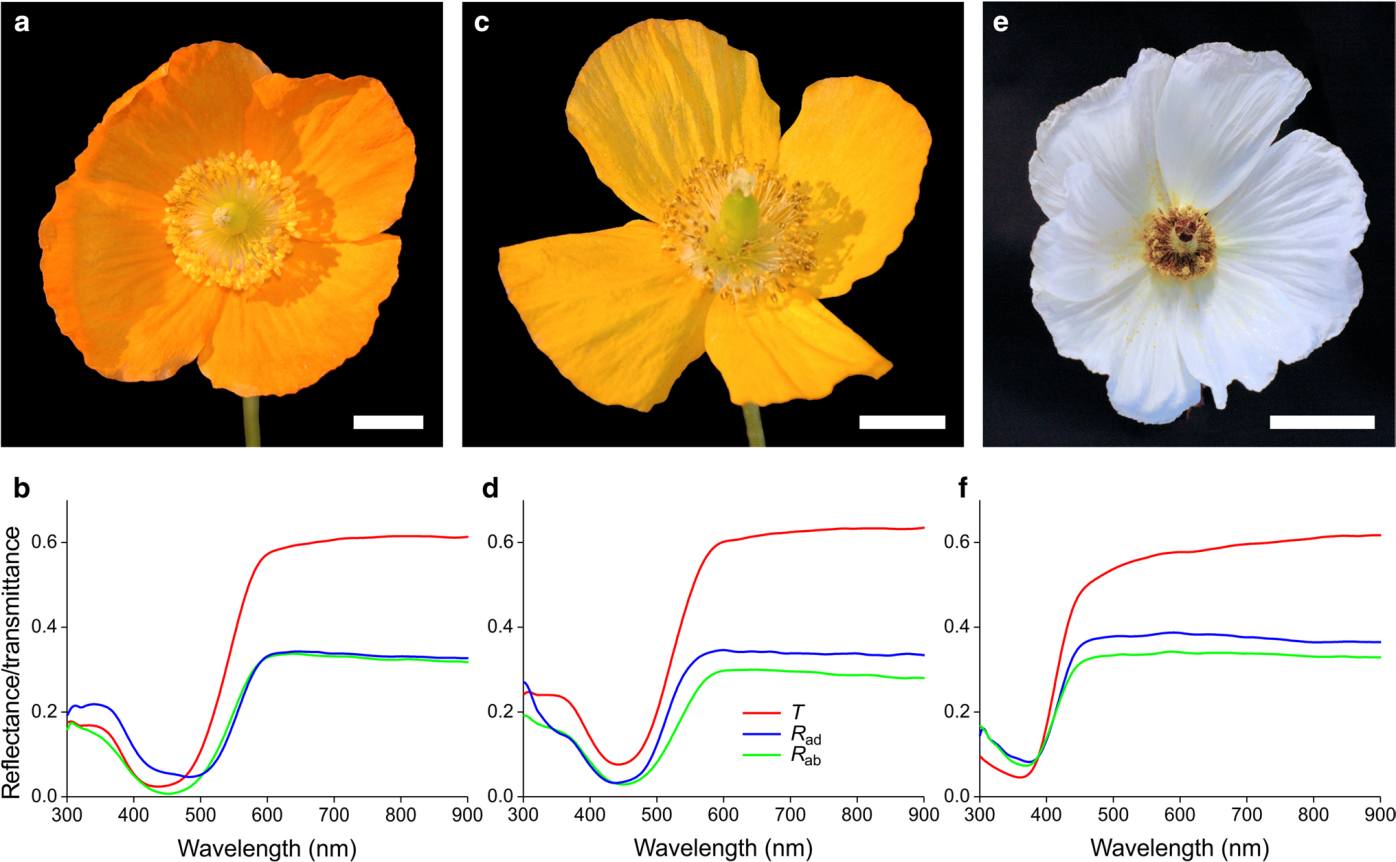
Flowers and spectral characteristics of the studied species. **a, c** Yellow and orange type *Meconopsis cambrica*. **e***Argemone polyanthemos*. **b, d, f** Transmittance (*T*) and adaxial (*R*_ad_) and abaxial (*R*_ab_) reflectance spectra **b** yellow *M. cambrica*; **d** orange *M. cambrica*; **f***A. polyanthemos*. Scale bar **a, c**, 1 cm; **e** 2 cm

The long-headed poppy (*P. dubium*) displays similarly beautiful red flowers, although this species rarely has black basal areas (Fig. 1d). The spectral slopes of the distal and proximal spectra in the long wavelength range are somewhat similar, but the spectral values in the ultraviolet measured at the proximal area are clearly lower than those for the distal area, indicating different amounts of ultraviolet-absorbing pigment (Fig. 1e,f). The Welsh poppy (*M. cambrica*) has an orange (Fig. 2a) and a yellow morph (Fig. 2b), and their transmittance and reflectance spectra are similar for all petal areas (Fig. 2c, d). The valleys in the spectra indicate the presence of a pigment absorbing mainly in the blue wavelength range. The crested prickly poppy (*A. polyanthemos*) has white flowers that are somewhat larger than for other species. The valley in the spectra of *A. polyanthemos* indicate a pigment absorbing exclusively in the ultraviolet wavelength range. For *A. polyanthemos*, there were no differences in reflectance between different flower areas.

Ultraviolet photography showed that there are no great differences in coloration of the petals, but the flowers as a whole feature a contrasting ultraviolet pattern. Flower patterns with ultraviolet-absorbing centres and ultraviolet-reflecting peripheries are known as “bulls-eye patterns”, due to the absence of ultraviolet reflection of the anthers and stigma (Fig. S1).

### Pigmentation and scattering is localised

**Fig. 3:**
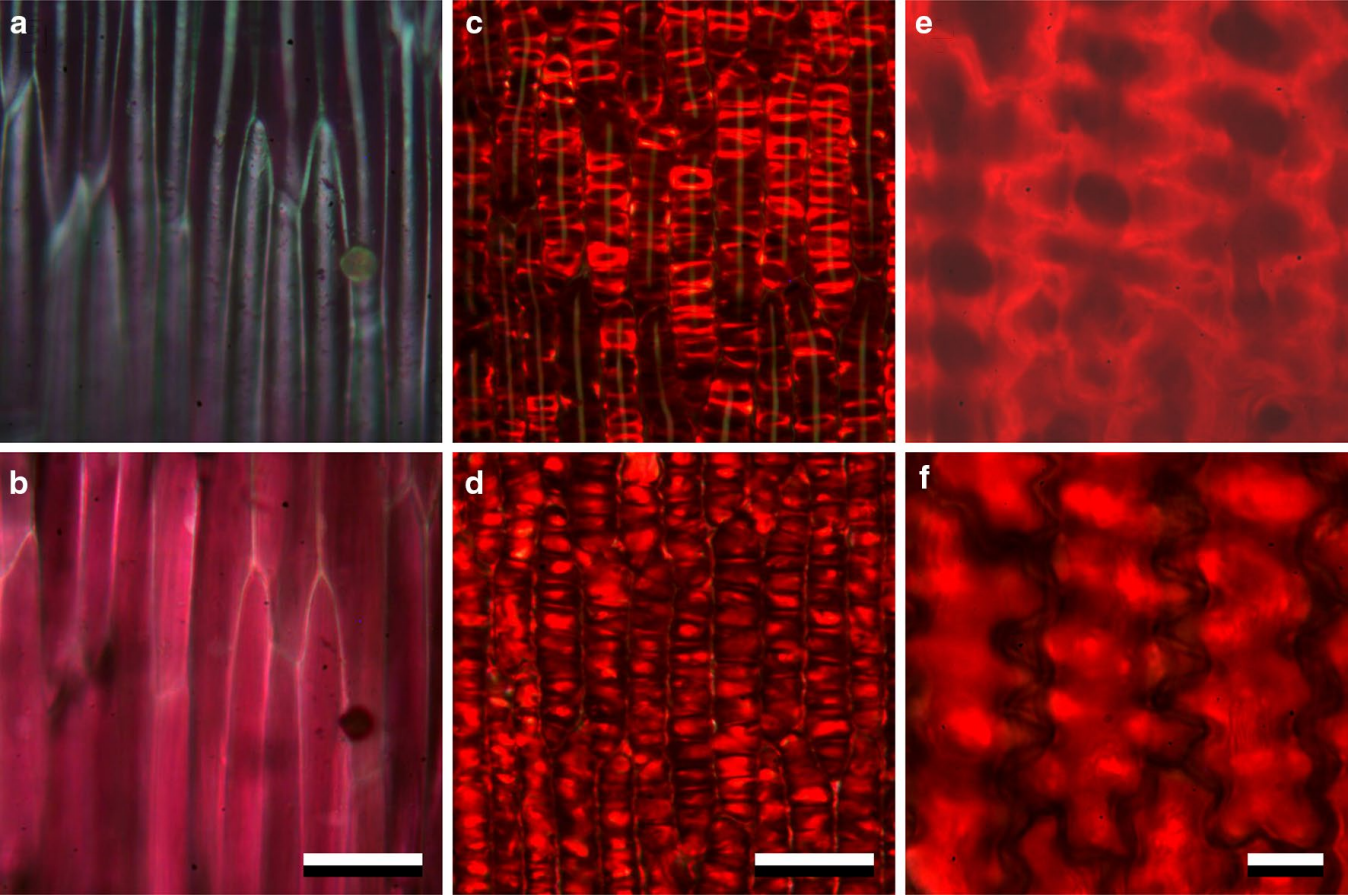
*P. rhoeas* petal areas observed close up (all top view). **a, b** Proximal area. **c**–**f** Distal areas. **a**–**d** Petal pieces in air. **e, f** Petal piece immersed in oil. Top row, **a, c, e** images observed with epi-illumination. Bottom row, **b, d, f** images observed with transmitted light. Scale bars **a**–**d** 50 µm; **e, f** 10 µm

The transmittance and reflectance spectra of flowers depend on both the absorption of incident light by pigments and the scattering by the floral structures. We observed the petals at high magnification with our Zeiss microscope (“Materials and methods”), applying epi-illumination as well as transmitted light (Fig. 3). With epi-illumination, the faint linear surface reflections observed at the proximal part of a *P. rhoeas* petal reveal very elongated, convex cells (Fig. 3c). In line with reflectance spectra obtained for this black area (Fig. 1a, c), the images showed that backscattering in the visible wavelength range by the components inside the proximal cells is low (Fig. 3a). In transmitted light, the cells nevertheless appear purplish (Fig. 3b), corresponding to the non-negligible transmittance in the violet and red wavelength range (Fig. 1c).

The appearance of the distal petal areas is different. Epi-illumination also creates an assembly of faint, linear surface reflections, but the reflections from inside the petal are bright-red (Fig. 3c). In transmitted light, the cells are also red, but at locations complementary to those appearing with epi-illumination (Fig. 3d). The elongated epidermal cells seem to be compartmentalised (Fig. 3c, d), but the compartmentalisation is an optical illusion caused by the wavy cell walls. The petal’s wavy cell walls are more clearly observed in oil immersion: epi-illumination reveals serpentine-wavy cell boundaries that appear bright, whereas in transmitted light mostly the cell interior is brightest (Fig. 3e, f).

**Fig. 4:**
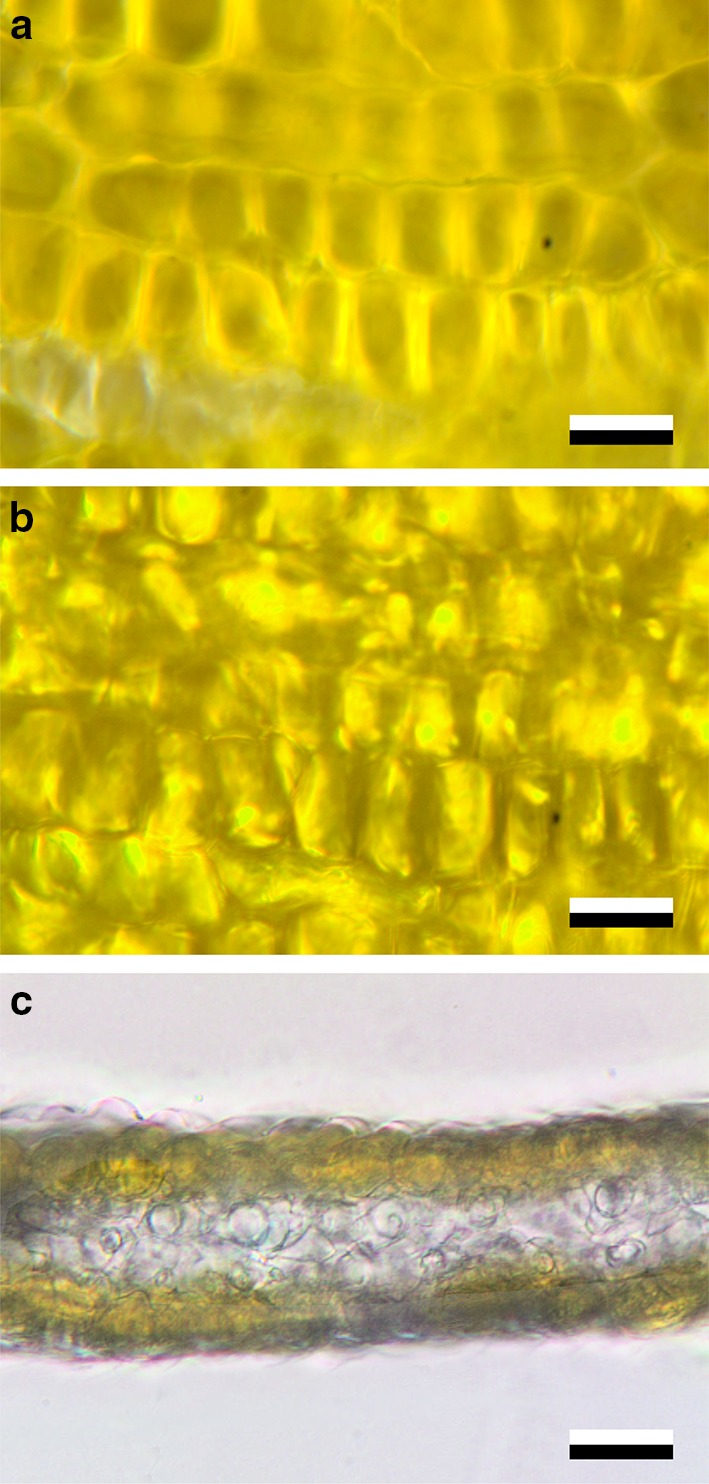
Structure of yellow *Meconopsis cambrica* flowers. **a** Close-up of epi-illuminated petal (top view). **b** The same area as in **a** trans-illuminated (top view). **c** Cross-section of the petal. Scale bars **a, b** 20 µm; **c** 50 µm

The flowers of *M. cambrica* feature a similar complementary behaviour of reflection and transmission (Fig. 4a, b). For yellow *M. cambrica* flowers, transverse sections furthermore revealed that the short-wavelength absorbing, yellow transmittant pigment is located in only the upper and lower epidermal cells, whereas the mesophyll layer in between is unpigmented (Fig. 4c). A similar epidermal deposition of pigment has been described for *P. rhoeas* (Kay et al. 1981) and *M. grandis* (Yoshida et al. 2006), and is typical for anthocyanins (Kay et al. 1981; Lee 2007).

**Fig. 5:**
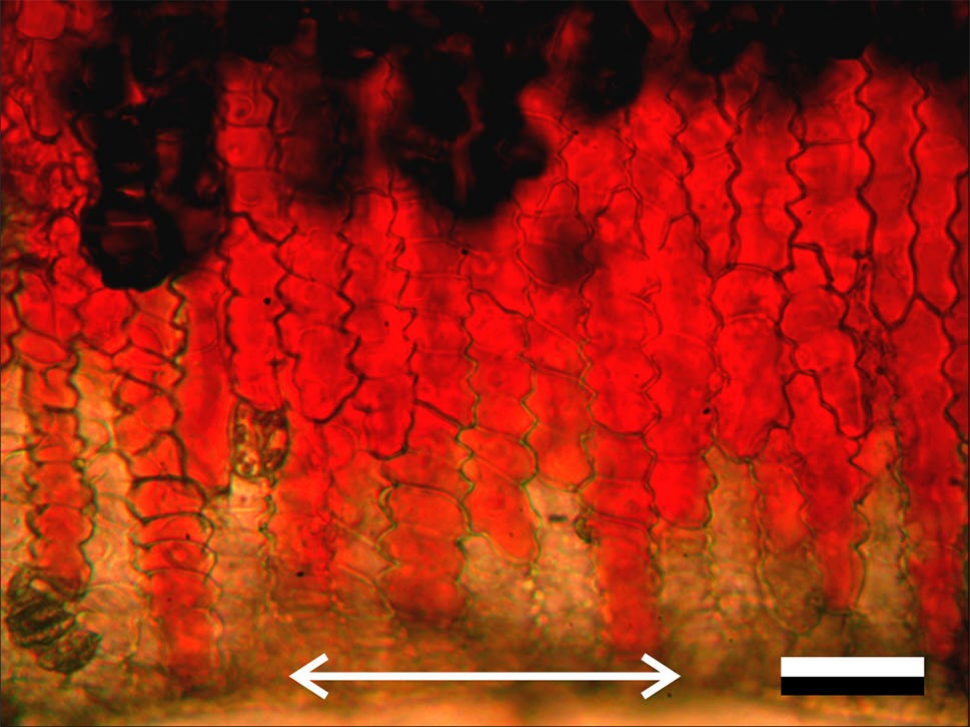
A cut *P. rhoeas* petal immersed in water with focus at the upper epidermis level in transmission. The cut area, where the pigment is gone, is indicated by the double-headed arrow; in the top part the air holes are still intact, causing the blackness. Scale bar 50 µm

The bright cell boundaries observed in epi-illumination versus the bright cell interior under transmitted light suggest the presence of scattering structures around and/or in between the (epidermal) cells. We investigated the presence of air cavities further by cutting a petal of a common poppy (*P. rhoeas*), about perpendicularly to the axes of the epidermal cells, and exposing the cut surface to water. Initially, the images were turbid (not shown), but starting from the cut surface the image clarified, and after a few minutes a clear image of the flower area resulted (Fig. 5). Figure S2 shows the same flower area at three different planes of focus: the upper epidermis, the interior, mesophyll layer, and the lower epidermis layer (Fig. S2a–c, respectively). The turbid area (Figs. 5, S2a–c, top) represents an area with strongly scattering air cavities, and thus appears dark under transmission. The elongated, homogeneous red coloured cells (Figs. 5, S2a–c, middle area) have become clearly visible, because the air cavities were filled with water by capillary action. The transected epidermal cells became transparent, due to leakage of the red colouring pigment (Figs. 5 double-headed arrow, S2a–c bottom).

### Pigments absorb exclusively in the ultraviolet or well into the red wavelength range

**Fig. 6:**
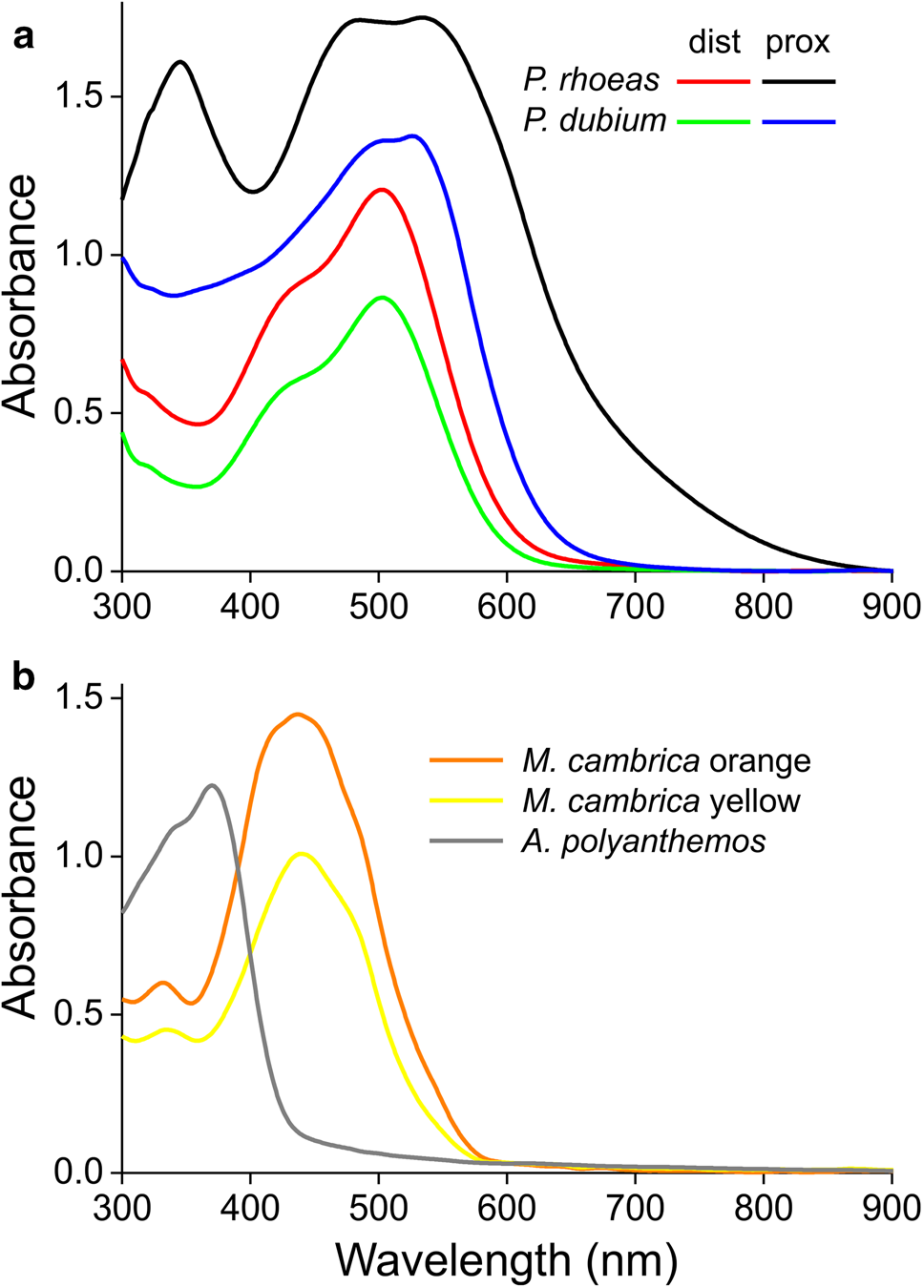
Absorbance of poppy petals. **a** Absorbance spectra of distal and proximal areas of *P. rhoeas* and *P. dubium* petals. **b** Absorbance spectra of petals of orange and yellow type *M. cambrica* and of *A. polyanthemos* petals

To obtain a closer view of the absorption properties of the various poppy pigments, we calculated the absorbance of the flowers shown in Fig. 1 (see “Materials and methods”). The absorbance spectra indicate that the distal areas of *P. rhoeas* and *P. dubium* petals contain the same pigment, with a lower density in *P. dubium* (Fig. 6a). The pigment in the proximal area of *P. rhoeas* is different, however, as it has a much higher absorbance tail in the red as well as an ultraviolet peak. The absorbance spectra of the orange and yellow *M. cambrica* are proportional, meaning that the two morphs feature the same pigment, but the amount is much higher in the orange morph. Finally, the absorbance spectrum of *A. polyanthemos* is almost fully restricted to the ultraviolet, which is typical for flowers that appear white to the human eye (Chittka et al. 1994; Kevan et al. 1996; van der Kooi et al. 2016).

**Fig. 7:**
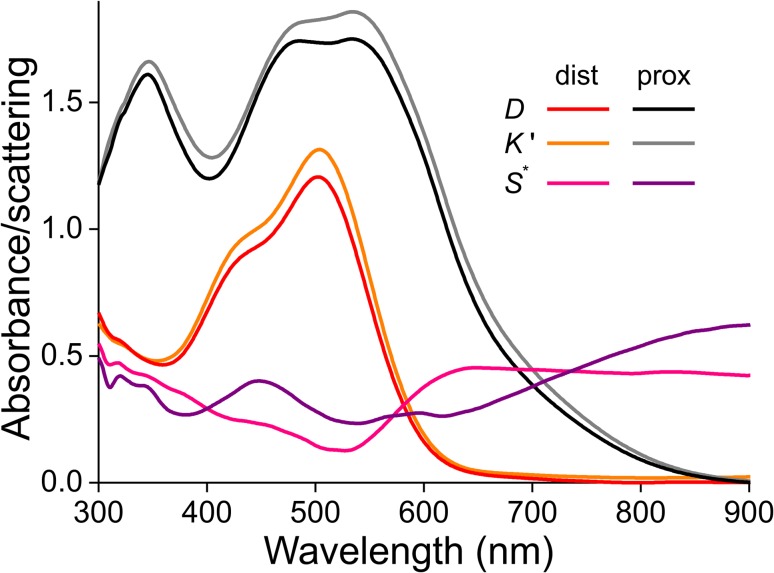
Absorbance and scattering in a *P. rhoeas* flower. The absorbance *D* of the distal (dist) and proximal (prox) part of the petal is identical to that of Fig. 6a. The parameter *K′* = 0.4343K^*^, where K^*^ is the absorption parameter that follows together with the scattering parameter S^*^ from the Kubelka–Munk layer-stack model analysis

The absorbance spectra of Fig. 6 represent only an approximation of the absorption properties of the various poppy pigments, because the different species of flower will have different scattering properties, especially given their markedly different thicknesses (Table 1). We previously developed an optical model that considers a flower as a stack of absorbing and scattering media, which we successfully applied to study the optics of the Chilean bellflower, buttercups and other flowers (Stavenga and van der Kooi 2016; van der Kooi et al. 2016, 2017). We applied the same model to *P. rhoeas* flowers and thus obtained the spectra of the absorption and scattering parameters for the distal and proximal petal parts (Fig. 7). The scattering parameters are clearly wavelength dependent, which is related to the absorption by the pigment. When we compare the experimental absorbance spectra with the modelled absorbance spectra, it appears that scattering has only a minor effect (for calculation procedures, see “Materials and methods”).

### Obliquely incident light causes polarised reflections

**Fig. 8:**
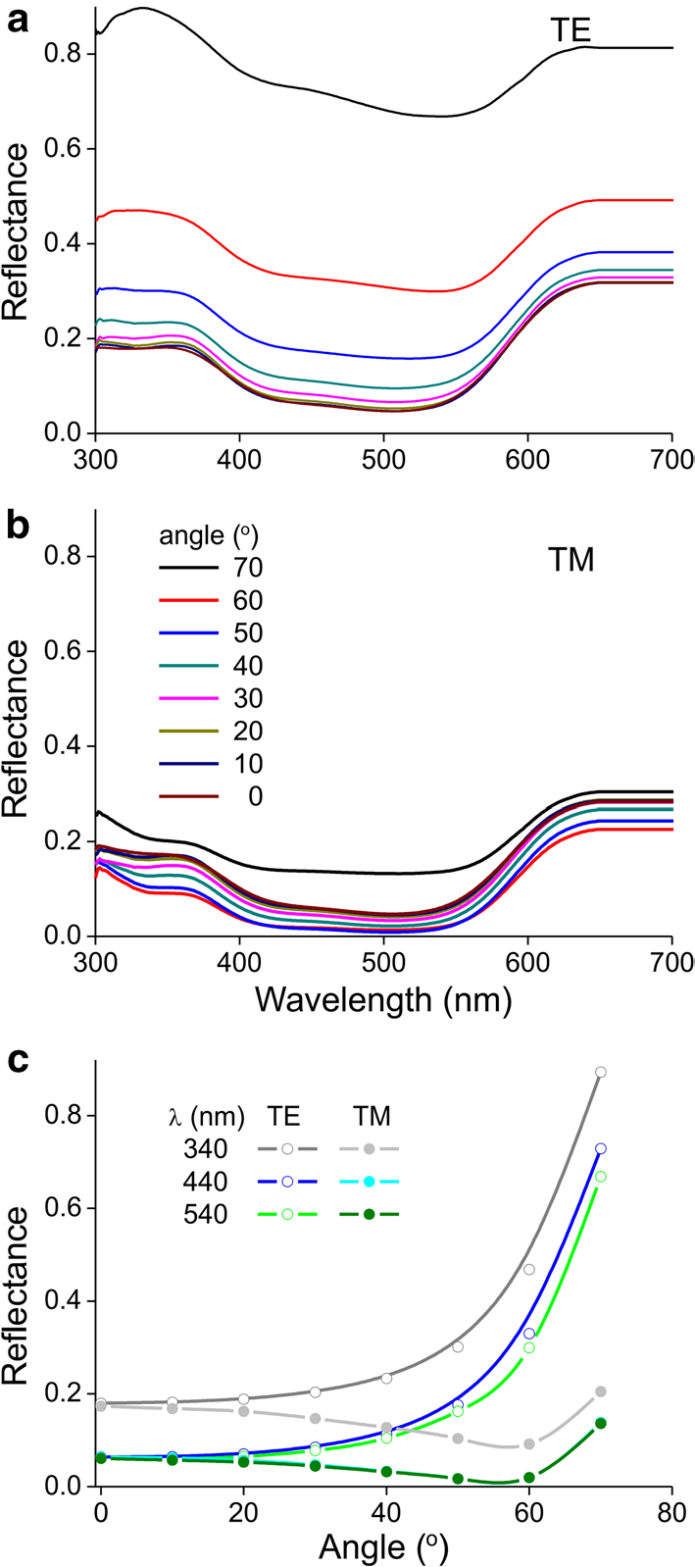
Angle-dependent reflectance of a *P. rhoeas* flower. **a** Reflectance spectra for TE-polarised light incident at angles 0, 10, …70°. **b** Reflectance spectra for TE-polarised light. **c** Reflectance values at wavelengths 340, 440 and 540 nm—which correspond to the peak sensitivities of the honey bee’s photoreceptors (Peitsch et al. 1992)—as a function of angle of light incidence

The reflectance spectra shown in Figs. 1 and 2 were measured for normal illumination, when surface reflection is generally very small (van der Kooi et al. 2014, 2015). Under large angles, however, surface reflection will increase, and when reflected by a smooth surface it becomes polarised (Wehner 2001). Indeed, under large angles relative to the normal, there are clear differences in reflectance amplitude between horizontally and linearly polarised light (Fig. 8a, b). In the blue and green wavelength ranges, the polarisation response of the petal was very similar to that of a water surface, with a Brewster angle at ~ 55° (Fig. 8c). For ultraviolet wavelengths, this was less distinct, but nevertheless oblique incident light causes significant polarisation.

## Discussion

Our study on the optical properties of poppy flowers revealed that they feature an interesting suite of optical and anatomical traits. *Papaver* flowers are exceedingly thin (Table 1), but contain a high amount of pigment and are fairly strong reflectors. Indeed, the (long-wavelength) reflectance value of 35% is high when considering their very minimal thickness (van der Kooi et al. 2016).

How can poppy flowers, with their average thickness of only three cell layers, be such efficient reflectors? The answer comes with the interior structure and shape of their cell walls. Microscopical investigation of individual cells showed that the serpentine-curved epidermal cell walls in combination with surrounding structures strongly scatter the light flux propagating in the petal interior, thus causing the bright appearance in epi-illumination and being dark in transmitted light (Figs. 3, 4). In addition, the interior air cavities are very prominent scatterers, because of the great difference in refractive index between the plant material and air. Immersion of a cut petal shows that the air cavities are gradually filled with water via capillary suction, thereby reducing the reflection (Fig. 5). Similar within-flower air cavities have previously been demonstrated to exist in flowers of *Caltha palustris* (Whatley 1984) and California poppy (Wilts et al. 2018), and have been shown to enhance the brilliance of buttercup flowers (Vignolini et al. 2012; van der Kooi et al. 2017).

Intriguingly, scattering does not increase with petal thickness, suggesting that the strong scattering structures only occur just below the epidermal cell layers in *Papaver* flowers. The mean thickness of flowers of *M. cambrica* and *A. polyanthemos* is significantly higher than the mean thickness of *P. rhoeas* and *P. dubium* (Table 1), but the long-wavelength reflectance values are similar (compare *R*_ad_ in Figs. 1, 2). An interesting open question remains as to how widespread the highly reflective typical cell structure is. The curvy cell walls occur in *Papaver* and *Meconopsis*, which are closely related (Kadereit et al. 2011; Xie et al. 2014), but we also observed similar cell shapes in the phylogenetically more diverged *A. polyanthemos* and *Chelidonium majus* (not shown). Hence, the typical cell shape—perhaps in thin petals in conjunction with the strongly scattering air cavities—may be widespread throughout Papaveraceae. Given the curvy cell walls also occur in leaves of many angiosperms (Vőfély et al. 2018), they are likely to occur widely in flowers too.

The studied poppy species feature a wide array of colours, due to various floral pigments, with absorption maxima for different species peaking over the whole visible wavelength range (Fig. 6). Furthermore, the proximal and distal parts of *P. rhoeas* and *P. dubium* flowers contain different pigments. The red, orange and yellow flower colours are likely due to anthocyanin pigments (Grotewold 2006; Ng and Smith 2016), though chemical investigations are required to determine the exact type of pigment. Our observations that the pigments are water-soluble and occur only in the epidermal cell layers (Figs. 4, 5) indeed suggest the presence of anthocyanin pigments. Flower colour polymorphisms, as seen in *M. cambrica*, also most commonly occur with anthocyanin-based colours (Narbona et al. 2018).

For flowers of *P. rhoeas*, there appears to be an ultraviolet colour polymorphism linked to the visual system of their pollinators. In Europe, flowers of *P. rhoeas* reflect ultraviolet light (Daumer 1958; Lunau 1993; van der Kooi et al. 2016; this study) and are pollinated by bees (McNaughton and Harper 1960; Proctor et al. 1997), but in Israel, the flowers lack ultraviolet reflection and are pollinated by beetles (Dafni et al. 1990). Given that bees exhibit little sensitivity in the red part of the spectrum and high sensitivity in the ultraviolet (e.g., Peitsch et al. 1992), and some important flower-visiting Mediterranean beetle species are sensitive in the red part of the spectrum (e.g., Martínez-Harms et al. 2012), the lack of ultraviolet reflection in the eastern Mediterranean seems to be a convergence for beetle pollination (Dafni et al. 1990). The chemical characteristics of the floral pigments in different geographic regions will shed light on how ultraviolet-absorbing pigments were lost when *P. rhoeas* expanded its geographic range to Europe.

In contrast to the natural variability in ultraviolet reflection, there is no significant variation in red coloration of the two *Papaver* species, suggesting that the red coloration is more developmentally constrained and/or it serves a relevant signalling function for bees also. Indeed, although bees are commonly thought to be insensitive to red colours, Chittka and Waser (1997) have shown that this is not the case. The spectral sensitivity of bees extends until at least 650 nm, and red poppy flowers feature noticeable reflection below that wavelength (Fig. 1). Interpretation of the colours with a bee subjective view showed that the red petals are strongly contrasting to a green background (Table 2). On the other hand, the green contrast, which is a (long-distance) visual signal used by some insect pollinators including bees (Giurfa et al. 1996; Spaethe et al. 2001; Hempel de Ibarra et al. 2014; van der Kooi et al. 2019), is very low for *P. rhoeas* and *P. dubium* (Table 2). Nonetheless, the fairly large floral display, the high colour contrast and the within-flower contrast in *P. rhoeas* and *P. dubium* may yield a visually strong signal. Furthermore, on a clear day and under oblique illumination, the surface reflection may enhance the (long-distance) visibility. Bees and other flower visitors may perceive the specular reflection as a flash, though it seems unlikely that any polarisation effect per se (Fig. 8) acts as a cue; for example, because the part of the bee eye that generally faces the flower is not polarisation-sensitive (for further discussion on polarised signalling by plants, see Wehner and Bernard 1993; van der Kooi et al. 2019).

**Table 2:** Vision modelling results

Species	Green contrast	Hexagon units	RNL units
*P. rhoeas*	0.04	0.23	7.3
*P. dubium*	0.01	0.23	6.8
*M. cambrica* yellow	0.21	0.18	7.3
*M. cambrica* orange	0.16	0.30	11.6
*A. polyanthemos*	0.33	0.21	9.7

Stimuli were compared against an average green backdrop, with D65 illuminant and spectral sensitivities for honey bees (see “Materials and methods”)

The differently coloured anthers, the dark red in the proximal part of the petal and—if present—the black markings furthermore create a within-flower colour contrast, making the centre of the flower where the pollen are located to visually stand out (Figs. 1, 2, S1). In addition to generating within-flower contrast, the black markings may increase attractiveness to pollinators by means of sexual deception. Experiments with artificial *P. rhoeas* “flowers” and pollinators in natural Israeli habitats showed that visitation by male beetles increases with black markings, presumably because males are looking for female beetles that often dwell in these flowers (Dafni et al. 1990). Similar results have been reported for the fly-pollinated South African daisy, *Gorteria diffusa* (Johnson and Midgley 1997; Ellis et al. 2014).

In summary, our study shows that despite their minimal thickness and floppy appearance, poppy flowers exhibit an intriguing anatomy. High pigment content, wavy cell walls and interior air spaces, combined with black markings and anthers create a conspicuous flower.

## Electronic supplementary material

Below is the link to the electronic supplementary material.

Supplementary material 1 (DOCX 263 KB)
