# Supplementary material for: Vividly coloured poppy flowers due to dense pigmentation and strong scattering in thin petals
Source: J Comp Physiol A Neuroethol Sens Neural Behav Physiol. 2019 Jan 28;205(3):363–72. doi: 10.1007/s00359-018-01313-1 (PMC6579775; doi:10.1007/s00359-018-01313-1)
Supplement: Supplementary file 1 — Supplementary material 1 (DOCX 263 KB) [file 359_2018_1313_MOESM1_ESM.docx]

**Supplementary material**


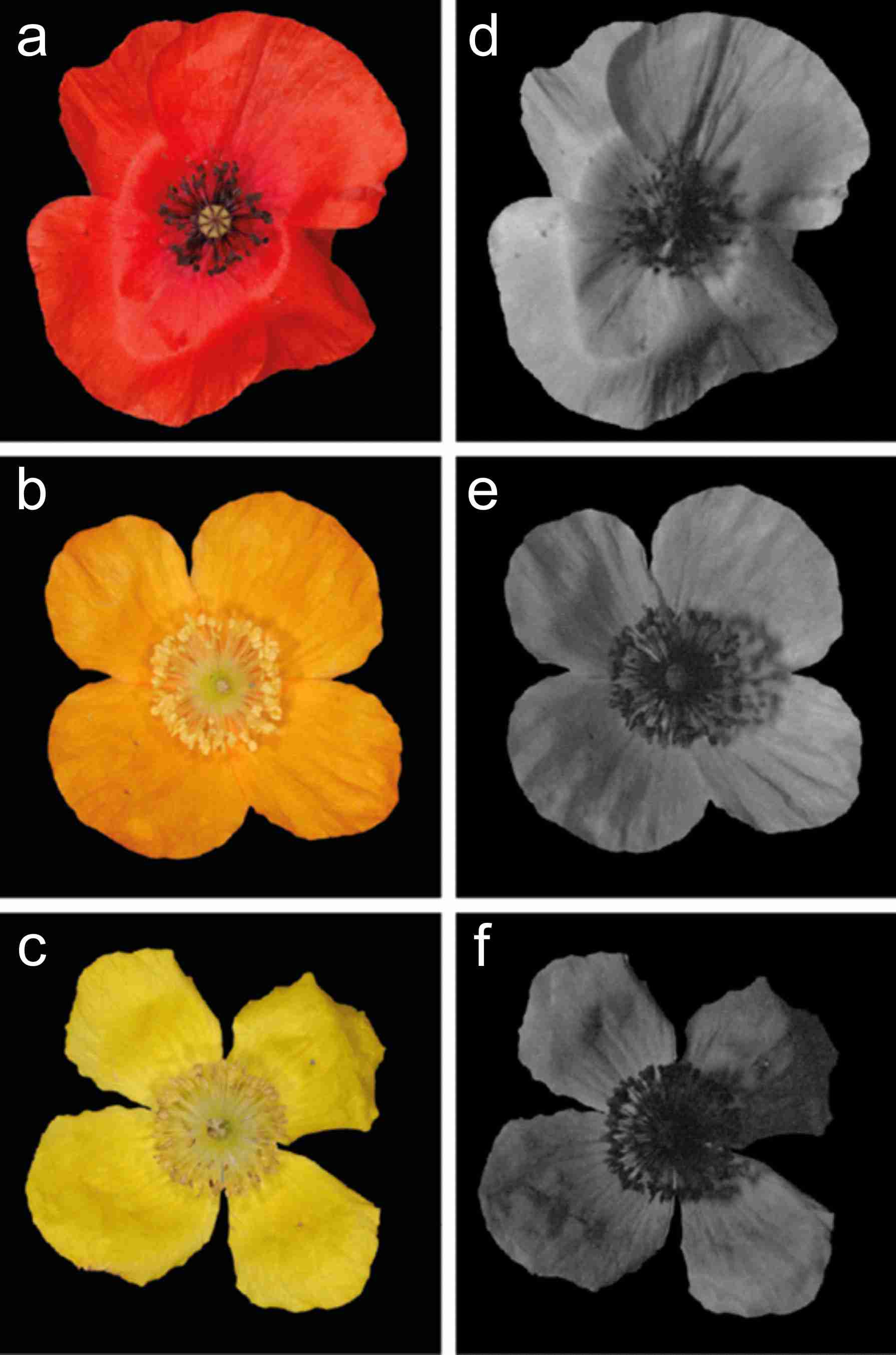


Figure S1. RGB (**a**-**c**) and ultraviolet (**d**-**f**) photographs of a *P. dubium* (**a**, **d**) flower and of orange (**b**, **e**) and yellow (**c**, **f**) *M. cambrica* flowers.


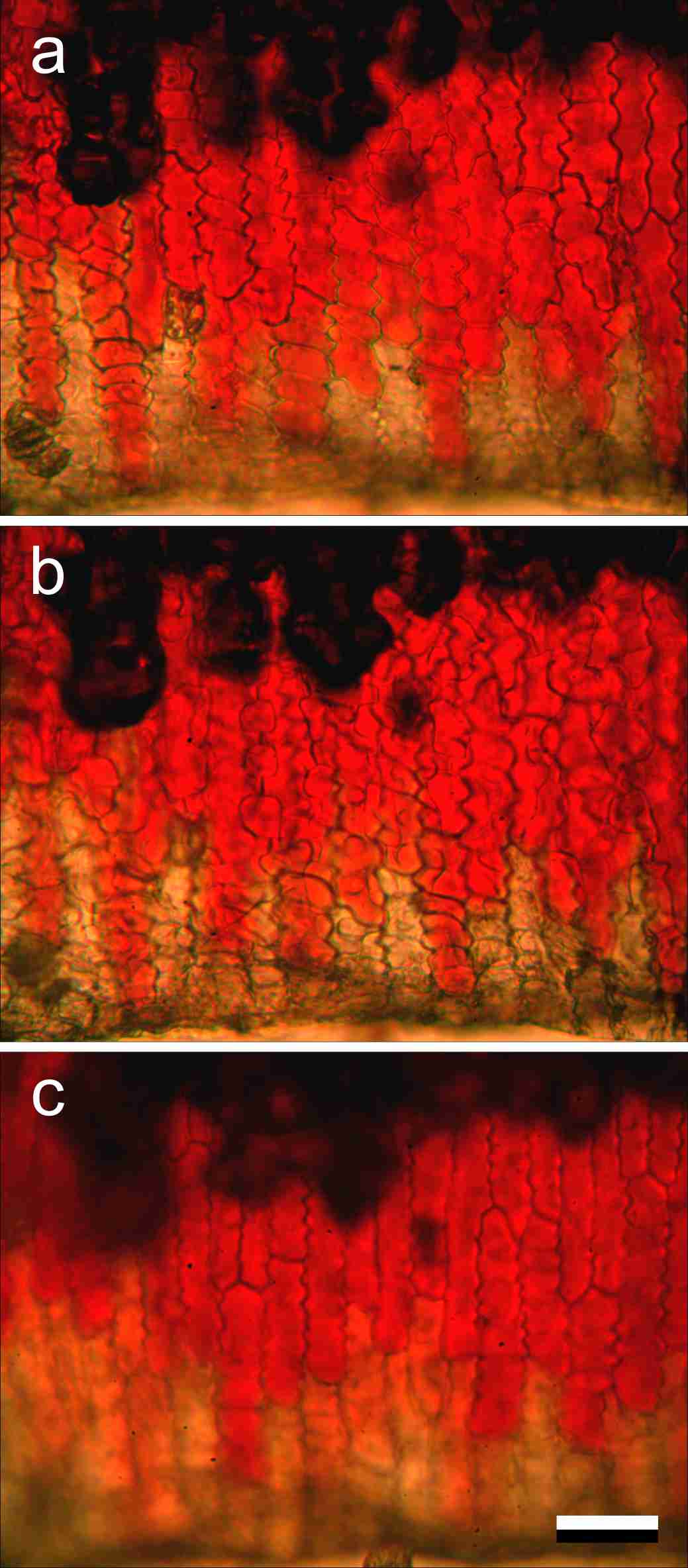


Figure S2. A cut *P. rhoeas* petal immersed in water, observed at different levels of focus (all in transmission). **a** Focus at upper epidermal layer. **b** Focus at intermediate level. **c** Focus at lower epidermal layer. The black area in the top of the images are air cavities that have not yet been filled with water and thus appear dark in the transmitted light. Scale bar: (**a**-**c**) 50 µm.
